# A Novel Protein NLRP12‐119aa that Prevents Rhabdovirus Replication by Disrupting the RNP Complex Formation

**DOI:** 10.1002/advs.202409953

**Published:** 2025-01-04

**Authors:** Weiwei Zheng, Xiangxiang Zhu, Tongtong Zhu, Qiang Luo, Yan Zhao, Tianjun Xu

**Affiliations:** ^1^ Laboratory of Fish Molecular Immunology College of Fisheries and Life Science Shanghai Ocean University Shanghai 201306 China; ^2^ Laboratory for Marine Biology and Biotechnology Qingdao Marine Science and Technology Center Qingdao 266200 China; ^3^ Marine Biomedical Science and Technology Innovation Platform of Lin‐gang Special Area Shanghai 201306 China

**Keywords:** circNLRP12, circRNA therapy, innate immunity, rhabdovirus, RNP

## Abstract

The accurate assembly of the ribonucleoprotein (RNP) complex is fundamental for the replication and transcription of rhabdoviruses, which are known for their broad pathogenic impact. A novel 119‐amino‐acid protein, NLRP12‐119aa is identified, encoded by the circular RNA circNLRP12, that effectively disrupts the formation of rhabdovirus RNP complexes through two distinct mechanisms and significantly reduces their replication. NLRP12‐119aa exhibits a strong affinity for the conserved 18‐nucleotide sequence at the start of the leader RNA of rhabdoviruses VSV, SCRV, and RABV, outcompeting their native N protein interactions, thereby disrupting the assembly of RNP complexes and inhibiting viral replication. NLRP12‐119aa exerts anti‐rhabdoviral effects by directly binding to the viral N protein, leading to its destabilization and accelerated degradation, and consequently hindering the formation of the viral RNP complex. To assess the therapeutic potential of circNLRP12 against rhabdovirus infections, a zebrafish model of VSV infection is established and noted a substantial reduction in viral load after‐treatment with circNLRP12, as well as the recovery of spleen's to a normalized state from its previously enlarged and hemorrhagic state. Collectively, these findings elucidate a novel dual anti‐RNP assembly strategy mediated by NLRP12‐119aa, offering valuable insights for further exploration and clinical management of rhabdoviral infections.

## Graphical abstract

The NLRP12‐119aa protein exerts its antiviral effect by two distinct mechanisms. First, it competes with the N protein of rhabdoviruses for binding to the viral leRNA, thereby preventing the formation of the RNP complex. Second, NLRP12‐119aa directly interacts with the rhabdovirus N protein, destabilizing it and promoting its degradation, which ultimately disrupts the assembly of the viral RNP complex.

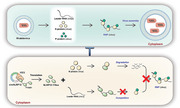

## 1 Introduction

Rhabdoviridae, a family of negative‐sense single‐stranded RNA viruses with a distinctive “bullet‐like” morphology, derives its name from this unique shape, which has significant implications for virological diagnosis and taxonomy.^[^ ^1^ ^]^ Rhabdoviridae encompasses six genera, the most extensively studied of which are Lysandviruses (prototype: rabies virus, RABV) and Vesiculovirus (prototype: vesicular stomatitis virus, VSV).^[^ ^2^ ^]^ VSV is renowned for its significant transmission capability, capable of infecting a broad range of organisms including mammals, fish, plants, and insects, with the distinctive ability to rapidly suppress the intrinsic mechanisms of host cells post‐infection, thereby promoting efficient self‐replication and significantly influencing pathogenic mechanisms and transmission strategies.^[^ ^3^ ^]^ RABV infection is primarily mammalian; without timely and effective prevention or treatment, it can cause acute and fatal central nervous system diseases, particularly in the developing countries of Asia, Africa, and Latin America, where it claims tens of thousands of lives annually.^[^ ^4^ ^]^ Thus, in‐depth studies on the infectious mechanisms of these viruses within Rhabdoviridae are of paramount significance.

Rhabdoviruses, one of the most diverse RNA virus families, infect a broad spectrum of hosts, including plants, vertebrates, and invertebrates, and occupy ecological niches across various environments, including marine, freshwater, and terrestrial ecosystems.^[^ ^2^, ^5^ ^]^ Rhabdoviruses can be transmitted through a multitude of pathways, including vector‐borne transmission and replication within arthropod vectors, plant sap, aerosols, animal bites, horizontal spread via contaminated water, and vertical transmission through the ova.^[^ ^6^ ^]^ The relatively conserved genetic structure of rhabdovirus, with genomes typically 11 to 16 kilobases in size and fixed order of at least five core genes (3′‐N‐P‐M‐G‐L‐5′), encodes the nucleoprotein (N), phosphoprotein (P), matrix protein (M), surface glycoprotein (G), and RNA‐dependent RNA polymerase (L), which collectively underpin the fundamental processes of rhabdovirus replication and assembly.^[^ ^7^ ^]^ Similar to many RNA viruses, the high mutation rates and genetic plasticity of rhabdoviruses pose significant challenges for diagnosis, treatment, and control.

Upon entering the cytoplasm through endocytosis, rhabdoviruses release infectious ribonucleoprotein (RNP) complexes that initiate the transcription and replication phases of their life cycle. The RNA polymerase recognizes and begins primary transcription of the RNP complex to produce an uncapped leader RNA (leRNA) and five mRNAs, which are then translated into the N, P, M, G, and L proteins essential for viral replication and assembly.^[^ ^2^, ^5^, ^8^ ^]^ The N and P proteins of rhabdoviruses bind to newly transcribed positive‐sense leRNAs, forming new positive‐sense RNP complexes that function solely as replication templates, devoid of the transcription signals present in the negative‐sense RNP complexes.^[^ ^2^, ^9^ ^]^ During replication, the N protein plays a pivotal role in packaging full‐length negative‐sense genomic RNA into novel RNP complexes, whereas the P protein serves as an auxiliary factor for the L protein, assisting in the scanning and processing of the RNA template to ensure accuracy and efficiency. The P protein safeguards the specificity and efficiency of viral replication by preventing the N protein from aberrantly assembling RNA chains that have not yet formed RNP complexes. The integrity and correct assembly of these RNP complexes are fundamental to the replication process^[^ ^5^, ^10^ ^]^ Our research aimed to explore the potential host pathways that may influence the formation of RNP complexes within the virus, using liquid chromatography‐mass spectrometry (LC‐MS) in order to identify proteins interacting with the leRNA strand of the VSV. Among the identified binding proteins, we discovered a novel protein with exceptionally high abundance, which was absent in existing mRNA transcriptome databases, leading us to expand our search to noncoding‐RNA databases and ultimately determine that it is a small‐molecule protein translated from circular RNA (circRNA).

Here, we characterized the novel 119‐amino‐acid protein NLRP12‐119aa, which is encoded by the exon‐intron circRNA circNLRP12 derived from the NLRP12 gene. Our research demonstrated a potent interaction between NLRP12‐119aa and leRNA of rhabdovirus, with its knockdown leading to a significant increase in viral replication. NLRP12‐119aa, composed solely of two leucine‐rich repeat (LRR) domains, can spontaneously assemble into a centrally symmetric multimeric structure, such as trimers, tetramers, and pentamers. Further research has confirmed the ability of NLRP12‐119aa to inhibit rhabdovirus replication through two distinct anti‐RNP assembly mechanisms and validated the therapeutic efficacy of circNLRP12 against rhabdovirus infections.

## 2 Results

### 2.1 Source and Functional Identification of NLRP12‐119aa

**Figure 1:**
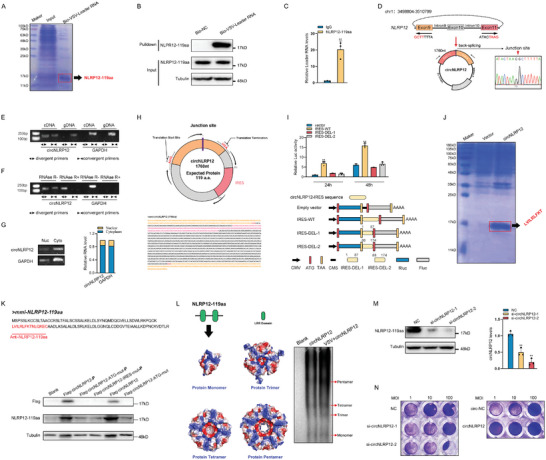
Source and functional identification of NLRP12‐119aa. A) RNA pull‐down combined with LC‐MS to detect proteins bound to VSV‐leRNA. Harvest the lysate from MKC cells and incubate it with biotinylated VSV‐leRNA. Subsequently, SDS‐PAGE electrophoresis will be employed to identify proteins that are selectively enriched by the VSV‐leRNA. B) RNA pull‐down verified VSV‐leRNA binds to NLRP12‐119aa. Use the NLRP12‐119aa antibody to detect whether NLRP12‐119aa protein can be enriched in MKC cells by biotinylated VSV‐leRNA (n = 3). C) Overexpression of NLRP12‐119aa in MKC cells, followed by detecting whether natural VSV‐leRNA can be enriched by NLRP12‐119aa protein using the NLRP12‐119aa antibody (n = 3). D) Confirmed head‐to‐tail splicing of circNLRP12 by Sanger sequencing. E,F) The expression of circNLRP12 and NLRP12 were detected by PCR and electrophoresis in the presence or absence of RNase R (n = 3). G) Nuclear cytoplasmic separation and PCR experiment to determine the distribution of circNLRP12 in cytoplasm and nucleus in MKC cells (n = 3). H) Distribution map and sequence of IRES and ORF regions of circNLRP12. I) IRES activity about circNLRP12‐IRES‐WT, circNLRP12‐IRES‐DEL‐1, and circNLRP12‐IRES‐DEL‐2 was tested in MSpC cells (n = 3). J) IP and LC‐MS detection of NLRP12‐119aa from circNLRP12. Overexpression of circNLRP12 in MSpC cells was detected for NLRP12‐119aa expression through SDS‐PAGE electrophoresis and LC‐MS. K) Flag‐tag and NLRP12‐119aa antibody was used to detect NLRP12‐119aa (n = 3). L) Protein structures of NLRP12‐119aa were predicted using Alpflod3 software, and use native‐PAGE electrophoresis to detect whether NLRP12‐119aa has an oligomeric form in MSpC cells (n = 3). M) Levels of NLRP12‐119aa and circNLRP12 after transfected with si‐circNLRP12 (n = 3). N) VSV viral titers were detected after transfected with si‐circNLRP12 in MKC cells with VSV infection or transfected with circNLRP12 in MSpC cells with VSV infection (n = 3). All data represent the means ± SE from three independent triplicate experiments. ^*^, *p* < 0.05; ^**^, *p* < 0.01.

The rhabdovirus family, renowned for its broad host range that infects plants, vertebrates, and invertebrates, causes incalculable economic losses in various breeding industries, underscoring the critical need for an in‐depth study of their evasion and host defense mechanisms^[^ ^5b,c^ ^]^ In this study, we have focused on the VSV to elucidate its resistance mechanisms against rhabdovirus, with a particular emphasis on identifying host defense strategies that may disrupt or interfere with the critical formation of the rhabdovirus RNP complex, which is essential for viral replication and transcription. We selected the VSV virus as the primary model for this study due to its extensive host range, which encompasses the ability to infect all vertebrates, from fish to mammals. This broad host adaptability renders VSV an exemplary model for investigating virus‐host interactions. In contrast, *Siniperca chuatsi* *Rhabdovirus* (SCRV) is predominantly restricted to lower vertebrates, particularly fish, whereas RABV is largely confined to higher mammals. Using RNA pulldown and LC‐MS, we successfully identified a novel, highly abundant protein, NLRP12‐119aa, which specifically binds to VSV‐leRNA in *Miichthys miiuy* kidney cells (MKC) (**Figure** **1**A). By synthesizing antibodies specific to NLRP12‐119aa (Figure 1K) and conducting additional RNA pulldown, an interaction with VSV‐leRNA was further elucidated, demonstrating the strong interaction between VSV‐leRNA and NLRP12‐119aa (Figure 1B). Using NLRP12‐119aa antibodies in RNA immunoprecipitation (RIP) assays, selective enrichment of VSV‐leRNA with the NLRP12‐119aa protein was confirmed (Figure 1C).

Identification of the origin of NLRP12‐119aa followed an unsuccessful search in standard mRNA transcript databases, which led to the examination of alternative transcriptomes and resulted in the discovery of its transcript sequence, circNLRP12, in the circRNA database. Derived from the NLRP12 gene located on chromosome 1, circNLRP12 is formed through the back‐splicing of exons 9–11, along with introns 9 and 10 of the NLRP12 gene, resulting in a circRNA of 1760 nucleotides (nt) (Figure 1D). circNLRP12 divergent primers were designed for RT‐PCR, and the amplified products were subjected to Sanger‐sequencing to confirm that circNLRP12 was spliced from head to tail (Figure 1D). Using convergent and divergent primers were used to, amplify the NLRP12 gene and circNLRP12, and performed RT‐PCR and electrophoresis were performed on cDNA and gDNA extracted from MKC, revealing exclusive amplification of circNLRP12 from cDNA and an absence of product from gDNA (Figure 1E). RNase R treatment validated the stability of circNLRP12 (Figure 1F) and subsequent nucleocytoplasmic separation experiments revealed its predominant cytoplasmic localization (Figure 1G).

The current understanding of circRNA translation mechanisms includes the IRES pathway and N6‐methyladenosine (m^6^A) modification pathway, and bioinformatics predictions suggest that circNLRP12 may possess a potential IRES sequence. The open reading frame (ORF) of circNLRP12 was been determined using the cORF pipeline software (Figure 1H). The prediction results showed that circNLRP12 encodes a protein composed of 119 amino acids, which we named NLRP12‐119aa (Figure 1H). To validate these aforementioned predictions, we assessed the IRES sequence activity of the predicted circNLRP12 and confirmed its capacity to mediate translation, which was significantly diminished by the deletion of the IRES sequence (Figure 1I). We detected the expression levels of NLRP12‐119aa in four cell lines, including MKC, *M. miiuy* spleen cells (MSpC), *M. miiuy* brain cells (MBrC), and *M. miiuy* liver cells (MLC). The results showed that MKC cells exhibited the highest expression, with MLC and MBrC showing intermediate levels, and MSpC cells displaying the lowest expression among the four (Figure S1D, Supporting Information). And VSV virus is more likely to infect the spleen and kidneys in fish. Therefore, in order to better validate the function of NLRP12‐119aa, we selected the MKC cells with the highest expression level and the MSpC cells with the lowest expression level for the following experiments. To further validate the coding potential of circNLRP12 for NLRP12‐119aa, overexpressed circNLRP12 in MSpC, immunoprecipitated the endogenous NLRP12‐119aa protein with NLRP12‐119aa antibodies, and characterized its amino acid sequence using LC‐MS, with results indicating the detectable amino acid sequence of the NLRP12‐119aa protein (Figure 1J).

In order to ascertain the coding capacity of circNLRP12, various overexpression plasmid mutants and circRNA variants were constructed, including plasmids such as circNLRP12‐P, Flag‐circNLRP12‐P, Flag‐CircNLRP12‐IRES‐mut‐P (lacking the IRES sequence), and Flag‐circNLRP12‐ATG‐mut‐P (lacking the “ATG” codon), as well as circRNAs like Flag‐circNLRP12 and Flag‐circNLRP12‐ATG‐mut (lacking the “ATG” codon), alongside circ‐NC (Figure 1K). Upon transfection of these plasmids and circRNAs into MSpC cells and subsequent Western blot analysis 48 h post‐transfection, the expression of the NLRP12‐119aa protein was detectable exclusively in samples with the Flag‐circNLRP12‐P plasmid, and in circRNA Flag‐circNLRP12, but was absent in those with the Flag‐circNLRP12‐ATG‐mut‐P, Flag‐circNLRP12‐IRES‐mut‐P, or circRNA Flag‐circNLRP12‐ATG‐mut, thereby confirming the NLRP12‐119aa protein coding potential of circNLRP12 dependent on the IRES pathway (Figure 1K). Predictive analysis indicated that the NLRP12‐119aa protein features only two LRR domains that are commonly associated with immune recognition, suggesting a pivotal role for this protein in the immune response (Figure 1L). Utilizing the Alpflod3 software, we predicted that NLRP12‐119aa protein monomers can spontaneously assemble into symmetrical, central hollow ring oligomeric forms—trimers, tetramers, and pentamers indicating a potentially critical role for the NLRP12‐119aa protein in biological processes because of its unique architecture (Figure 1L). Using native‐PAGE electrophoresis, we confirmed that NLRP12‐119aa exists in various oligomeric forms, aligning closely with our predicted outcomes (Figure 1L). Upon VSV virus infection, there is a notable increase in both the expression levels and the oligomeric forms of NLRP12‐119aa (Figure 1L), and this correlation suggests that NLRP12‐119aa's antiviral activity may be mediated through its oligomeric structures.

We designed two siRNAs targeting circNLRP12, si‐circNLRP12‐1, and si‐circNLRP12‐2, and initially assessed their knockdown efficiency upon their transfection into MKC using WB and qPCR, revealing si‐circNLRP12‐1 to have ≈60% efficiency and si‐circNLRP12‐2 to have ≈85% efficiency (Figure 1M). Subsequent experiments examining the impact of circNLRP12 knockdown on the VSV viral titer demonstrated that both siRNAs significantly increased the viral titer, with si‐circNLRP12‐2 exhibiting a more pronounced enhancing effect, especially after 12 h of infection (Figure 1N; Figure S1A, Supporting Information). And overexpression of NLRP12‐119aa can significantly reduce the titer of VSV viral (Figure 1N; Figure S1A, Supporting Information). Our analysis of NLRP12‐119aa's impact on VSV virus transcription revealed that its overexpression markedly suppressed the levels of VSV virus mRNA (N gene), whereas the knockdown of circNLRP12 led to a significant enhancement of VSV virus mRNA (N gene) levels (Figure S1B, Supporting Information). We found that overexpressing NLRP12‐119aa decreased the total RNA level of the VSV virus to 0.3 of its original amount, while the knockdown of circNLRP12 significantly increased the total RNA level of the VSV virus (Figure S1C, Supporting Information).

### 2.2 NLRP12‐119aa Disrupts the Assembly of the RNP Complex of VSV by Binding to VSV‐leRNA

**Figure 2:**
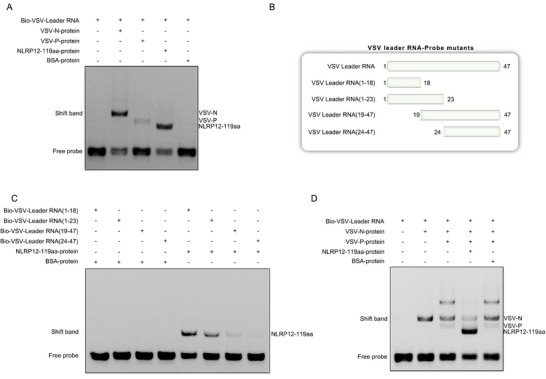
NLRP12‐119aa disrupts the assembly of the RNP complex of VSV by binding to VSV‐leRNA. A) RNA‐EMSA detects the interaction between biotinylated VSV‐leRNA and VSV‐N, VSV‐P, or NLRP12‐119aa protein, respectively (n = 3). B,C) RNA‐EMSA detects the interactions between various truncated mutants of biotinylated VSV‐leRNA and VSV‐N protein or NLRP12‐119aa protein (n = 3). D) RNA‐EMSA detects the effect of NLRP12‐119aa protein on the formation of VSV‐RNP complex (n = 3). All data represented the three independent triplicated experiments.

To further confirm the interaction between the NLRP12‐119aa protein and VSV‐leRNA, we performed an RNA electrophoretic mobility shift assay (EMSA) with in vitro transcribed biotin‐labeled full‐length VSV‐leRNA. This reveals a robust binding affinity not only between the VSV‐N protein and VSV‐leRNA but also an even stronger interaction between NLRP12‐119aa and VSV‐leRNA when compared to the VSV‐N protein, with the VSV‐P protein showing no direct binding to VSV‐leRNA (**Figure** **2**A). To identify the binding site of NLRP12‐119aa on VSV‐leRNA, we designed mutated probes and performed additional RNA‐EMSA, which indicated a strong interaction with the VSV‐leRNA segments (1–18) and, (1–23) and revealed that the primary binding site for NLRP12‐119aa was within the first 18 nt, given the significantly weaker binding affinity for other regions (Figure 2B,C). RNA‐EMSA conducted with a mixture of NLRP12‐119aa, VSV‐N, and VSV‐P proteins along with the VSV‐leRNA probe, demonstrated that the presence of NLRP12‐119aa increased the binding of the probe to NLRP12‐119aa and significantly decreased binding at the VSV‐N protein site, indicating that NLRP12‐119aa can disrupt the formation of the VSV RNP complex by outcompeting VSV‐N for binding to VSV‐leRNA (Figure 2D).

### 2.3 NLRP12‐119aa Disrupts the Assembly of the RNP Complex of VSV by Degrading the VSV‐N Protein

**Figure 3:**
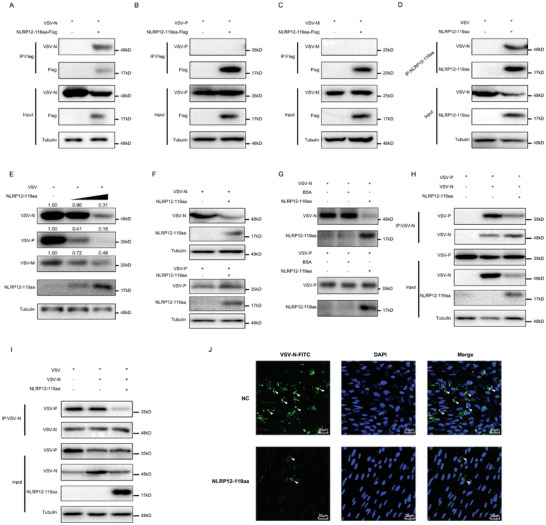
NLRP12‐119aa disrupts the assembly of the RNP complex of VSV by degrading the VSV‐N protein. A–C) IP analysis of the interaction between NLRP12‐119aa and VSV‐N (A) or VSV‐P (B) or VSV‐M (C) protein in MSpC cells (n = 3). D) IP analysis of the interaction between endogenous NLRP12‐119aa and VSV‐N protein in MKC cells (n = 3). E) Effect of NLRP12‐119aa on endogenous N, P, and M protein levels. Using antibodies specific to VSV‐N, VSV‐P, and VSV‐M to detect the levels of N, P, and M proteins in the VSV virus following the overexpression of the NLRP12‐119aa protein in MSpC cells (n = 3). F) Effect of NLRP12‐119aa on exogenous VSV‐N and VSV‐P proteins in MSpC cells (n = 3). G) Detected the effect of purified NLRP12‐119aa protein on VSV‐N and VSV‐P protein. H) IP analysis of the effect of NLRP12‐119aa on the interaction between exogenous VSV‐N and VSV‐P protein (n = 3). I) IP analysis of the effect of NLRP12‐119aa protein on the interaction between endogenous VSV‐N and VSV‐P protein in MSpC cells (n = 3). J) Immunofluorescence analysis of the effect of NLRP12‐119aa on the budding of VSV virus in MSpC cell (n = 3). All data represented the three independent triplicated experiments.

We investigated the impact of the NLRP12‐119aa protein on the structural proteins N, P, and M of VSV using IP with Flag‐antibodies targeting NLRP12‐119aa‐Flag, revealing a specific interaction with the VSV‐N protein (**Figure** **3**A), but no interactions with the VSV‐P and VSV‐M proteins (Figure 3B,C). We validated the interaction between NLRP12‐119aa and the VSV‐N protein through endogenous IP (Figure 3D). Further WB analysis revealed that NLRP12‐119aa significantly reduced the levels of VSV‐N, P, and M proteins (Figure 3E), since NLRP12‐119aa binding to VSV‐leRNA disrupts viral replication and transcription, the observed reduction might not be a direct effect on these proteins but rather a consequence of the overall suppression of viral replication. To clarify the mechanism underlying this phenomenon, further investigations are warranted to distinguish between the direct impact of NLRP12‐119aa on the stability or function of VSV proteins and the indirect effect resulting from its interference with viral replication at the leRNA level. After co‐transfecting MSpC cells with VSV‐N or VSV‐P plasmids along with an NLRP12‐119aa overexpression plasmid, we observed that NLRP12‐119aa markedly suppressed the expression of exogenous VSV‐N protein, with minimal impact on VSV‐P (Figure 3F). In vitro experiments, we purified and incubated NLRP12‐119aa, BSA, VSV‐N, and VSV‐P proteins at 37 °C for 30 min, and WB analysis revealed a significant increase in the degradation rate of VSV‐N when co‐incubated with NLRP12‐119aa, whereas VSV‐P remained unaffected, and BSA protein does not degrade VSV‐N protein, which indicates that NLRP12‐119aa may have a proteasome‐like effect or be able to disrupt the stability of VSV‐N protein (Figure 3G).

To determine whether NLRP12‐119aa's degradation effect on the VSV‐N protein influences VSV RNP complex formation, we assessed its impact on VSV‐N and VSV‐P protein interactions. Here, we are examining the impact of the NLRP12‐119aa protein on the viral RNP complex under conditions where the N protein level is held constant, focusing specifically on its influence on the P protein's binding capacity. However, the overexpression of NLRP12‐119aa can markedly diminish the N protein levels. Consequently, without proper controls, it is challenging to discern whether a reduction in P protein enrichment in IP samples is due to a decrease in N protein or due to NLRP12‐119aa disrupting the RNP complex. Both a decrease in N protein and the disruption of RNP complex formation by NLRP12‐119aa could lead to reduced P protein enrichment. To distinguish between these possibilities, we opted to adjust the IP loading amounts to assess P protein enrichment at equivalent N protein levels, ensuring that any observed changes are attributable to the effects of NLRP12‐119aa on the RNP complex rather than variations in N protein expression. Our findings indicated that NLRP12‐119aa significantly dampened the interaction between VSV‐N and VSV‐P proteins at equivalent enrichment levels (Figure 3H). NLRP12‐119aa also markedly decreased interactions between endogenous VSV‐N and VSV‐P proteins (Figure 3I). Through immunofluorescence staining of the VSV virus, we observed an intriguing phenomenon in which various‐sized round vesicular structures, seemingly indicative of the viral budding process, appeared outside the cells after infection, with NLRP12‐119aa significantly inhibiting their formation (Figure 3J).

### 2.4 NLRP12‐119aa Disrupts Rhabdovirus RNP Complex Assembly by Binding to the First 18nt of the leRNA

**Figure 4:**
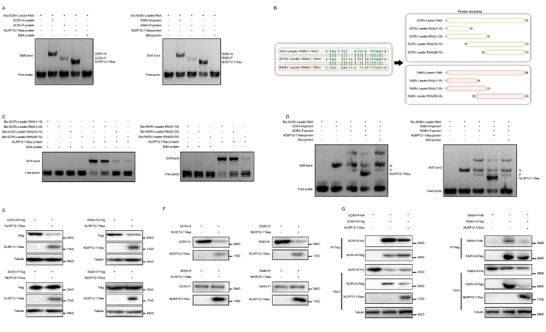
NLRP12‐119aa disrupts rhabdovirus RNP complex assembly by binding to the first 18nt of the leRNA. A) Detects interaction between leRNA and N protein with NLRP12‐119aa. RNA‐EMSA detects the interaction between biotinylated SCRV‐leRNA and SCRV‐N, SCRV‐P, or NLRP12‐119aa protein, respectively; RNA‐EMSA detects the interaction between biotinylated RABV‐leRNA and RABV‐N, RABV‐P, or NLRP12‐119aa protein, respectively (n = 3). B) Design of truncated mutants of leRNA in SCRV and RABV. C) RNA‐EMSA detects the interaction between truncated mutants of biotinylated SCRV‐leRNA and NLRP12‐119aa protein; RNA‐EMSA detects the interaction between truncated mutants of biotinylated RABV‐leRNA and NLRP12‐119aa protein (n = 3). D) RNA‐EMSA detects the effect of NLRP12‐119aa on the formation of SCRV‐RNP or RABV‐RNP complex (n = 3). E) Effect of NLRP12‐119aa on exogenous SCRV‐N and SCRV‐P proteins in HEK293 cells; Effect of NLRP12‐119aa on exogenous RABV‐N and RABV‐P proteins in BSR cells (n = 3). F) Detection of the effect of purified NLRP12‐119aa on virus N and P protein (n = 3). G) IP analysis of the effect of NLRP12‐119aa on the interaction between exogenous virus N and P protein in HEK293 cells (n = 3). All data represented the three independent triplicated experiments.

To determine whether the inhibitory mechanism of NLRP12‐119aa is specific to VSV or broadly applicable to other rhabdoviruses, we conducted experiments with SCRV and RABV, similar to those previously performed, and the results showed that NLRP12‐119aa could significantly inhibit the proliferation and transcription of SCRV and RABV viruses (Figure S1E, Supporting Information), and significantly reduce the total RNA content of both viruses (Figure S1G, Supporting Information). The initial assessments demonstrated that NLRP12‐119aa interacts with both SCRV‐leRNA and RABV‐leRNA, indicating a potentially broad mechanism of action (**Figure** **4**A). The selection of SCRV and RABV for validation was strategic. SCRV specifically infects lower vertebrates, RABV targets mammals, and VSV can infect invertebrates, thereby covering the antiviral efficacy of NLRP12‐119aa across the spectrum of rhabdoviruses affecting invertebrates to mammals. Sequence alignment revealed a high degree of conservation in the first 18–19 nt of the leRNA among SCRV, VSV, and RABV, leading us to hypothesize that, similar to VSV‐leRNA, the binding sites for SCRV‐leRNA and RABV‐leRNA with NLRP12‐119aa would be the initial 18–19 nt (Figure 4B). This hypothesis was confirmed through RNA‐EMSA using mutated probes for SCRV‐leRNA and RABV‐leRNA, demonstrating that NLRP12‐119aa exhibits a significantly higher binding affinity for the first 18 or 19 nt of leRNA compared to other regions (Figure 4C).

NLRP12‐119aa was found to disrupt the binding between SCRV‐leRNA and SCRV‐N protein as well as between RABV‐leRNA and RABV‐N protein, and similar to its effects on VSV (Figure 4D), it significantly promotes the degradation of both SCRV‐N and RABV‐N proteins in both in vitro and in vivo (Figure 4E,F). IP experiments demonstrated that NLRP12‐119aa markedly inhibited the interactions between N and P proteins in both SCRV and RABV (Figure 4G). Collectively, these findings indicate that the inhibitory mechanisms of NLRP12‐119aa, previously observed against VSV, are applicable to this virus but also to other rhabdoviruses, suggesting a broad‐spectrum antiviral response against this viral family.

### 2.5 CircRNA Therapy of circNLRP12 Can Effectively Treat the Infection of VSV in Zebrafish

**Figure 5:**
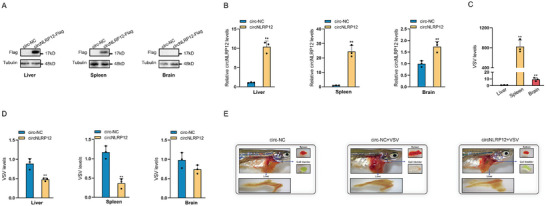
CircRNA therapy of circNLRP12 can effectively treat the infection of VSV in zebrafish. A,B) WB and qPCR were used to detect the expression of NLRP12‐119aa protein (A) and circNLRP12 (B) in liver, spleen, and brain tissues in zebrafish (n = 3). C) Levels of VSV mRNA (N gene) in the liver, spleen, and brain tissues were detected by qPCR in zebrafish with VSV (n = 3). D,E) Levels of VSV mRNA in tissues were detected by qPCR in zebrafish (D); Capture photos of the tissues for detailed observation (E) (n = 3). All data represent the means ± SE from three independent triplicate experiments. ^*^, *p* < 0.05; ^**^, *p* < 0.01.

To evaluate the potential of NLRP12‐119aa in treating rhabdovirus infections, we conducted experiments in a zebrafish model using an in vitro synthesized circNLRP12‐based circRNA therapeutic, injected synthetic circNLRP12 and Lipofectamine 3000 into the peritoneal cavity of zebrafish and collected tissues 48 h later for subsequent analyses. Both circ‐NC (control circRNA) and Flag‐circNLRP12 were injected into the peritoneal cavity of zebrafish, and after 48 h, the liver, spleen, and brain tissues were collected in order to evaluate the expression of the NLRP12‐119aa protein, finding that Flag‐circNLRP12 specifically induced its expression in the spleen and liver, but not in the brain (**Figure** **5**A), and increased circNLRP2 expression ≈25‐fold in the spleen, 10‐fold in the liver, and doubled in the brain (Figure 5B). Virus mRNA detection indicated that the VSV virus predominantly infected the zebrafish spleen, with a viral load over 800 times higher than in the liver and more than 80 times higher than that in the brain (Figure 5C), which correlated with gross pathological observations of VSV‐infected zebrafish, including splenic hemorrhage and enlargement, a change in gallbladder color from green to transparent, and minimal liver changes (Figure 5E). Assessment of NLRP12‐119aa's impact on VSV load revealed a significant reduction in the spleen and a moderate decrease in the brain (Figure 5D). Zebrafish treated with circNLRP12 exhibited robust protection against VSV infection, characterized by the preservation of the normal green hue of the gallbladder and the absence of splenic hemorrhage and swelling (Figure 5E).

## 3 Discussion

CircRNAs, a class of distinctive non‐coding RNA molecules formed by the covalent closure of loops through exon or intron back‐splicing, were initially regarded as incapable of encoding proteins but have recently been shown to possess the potential to encode functional proteins.^[^ ^11^ ^]^ Current research has revealed two principal mechanisms by which circRNAs can encode proteins: one involves the use of IRES to initiate translation, as demonstrated by circZNF609, circMbl, and circFGFR1,^[^ ^7^, ^12^ ^]^ and the other is m^6^A modification, which potently induces translation, including Rnf103‐177aa and MIB2‐134aa, where m^6^A modification enhances the translation process.^[^ ^13^ ^]^ Here, we identified a novel circRNA, circNLRP12, which mediates the translation of a 119‐amino‐acid protein, NLRP12‐119aa, via the IRES‐dependent pathway, its knockdown significantly was found to have enhanced VSV replication, implying a critical role for this protein in the host's antiviral immunity. Our research demonstrated that NLRP12‐119aa, with its high affinity for the leRNA strand of VSV, potently inhibits the formation of VSV RNP complexes by outcompeting the viral N protein for leRNA binding, suppressing VSV replication. This extends to other rhabdoviruses, as evidenced by the strong binding of NLRP12‐119aa to the leRNA of SCRV and RABV, suggesting its broad ability to disrupt RNP complex assembly. Sequence analysis identified a highly conserved region within the initial 18 nt of rhabdovirus leRNAs. By constructing mutants of this segment, we demonstrated that NLRP12‐119aa possesses a significantly enhanced binding affinity for these 18 nt, empowering it to impede RNP complex assembly and, by extension, curtail viral replication through its interaction with the conserved leRNA sequence. Further research indicates that NLRP12‐119aa selectively interacts with the N protein of rhabdovirus, which is unaffected by the P or M proteins, and efficiently promotes the degradation of the N protein both in vitro and in vivo, destabilizing it to prevent rhabdovirus RNP complex formation and inhibit rhabdovirus replication. This demonstrates that NLRP12‐119aa can impede the formation of rhabdovirus RNP complexes and suppress rhabdovirus replication via two distinct mechanisms.

Despite its compact size of 119 amino acids, NLRP12‐119aa significantly inhibits rhabdovirus replication and transcription by competing with the viral N protein to bind to the leRNA strand, which is a critical step in the formation of infectious RNP complexes, thereby neutralizing viral replication and transcription capabilities and countering their immune evasion strategies. NLRP12, a key innate immune receptor of the NLR family distinguished by its LRR domains essential for immune recognition, features ten such domains in its full‐length form,^[^ ^14^ ^]^ the shorter NLRP12‐119aa variant, composed of only 119 amino acids, retains two LRR domains, suggesting that it may significantly contribute to immune responses because of its preserved immune function‐associated LRR motifs. Employing the Alpflod3 software for structural prediction, we discovered that NLRP12‐119aa monomers self‐assemble into multimeric structures such as trimers, tetramers, and pentamers, which are centrally symmetric and form hollow ring‐shaped complexes, indicating the potential of the protein for critical biological functions.

Assessing the therapeutic efficacy of NLRP12‐119aa against rhabdovirus infection, we established a VSV‐infected zebrafish model and delivered circNLRP12 for circRNA therapy, with results showing that, NLRP12‐119aa significantly inhibits VSV replication in the zebrafish spleen and markedly improves virus‐induced splenic hemorrhage, supporting the potential of circNLRP12‐based treatment as a viable approach against rhabdovirus. Conclusively, our study reveals the novel protein NLRP12‐119aa's dual anti‐RNP assembly mechanisms and confirms the efficacy of circRNA therapy in treating organ hemorrhage caused by rhabdovirus infection, it provides not only new insights for developing therapeutic strategies against rhabdovirus diseases but also substantiating the potential of circRNA therapeutics.

## 4 Experimental Section

### Ethics Statement

Animal experimental procedures were performed in accordance with the National Institutes of Health's Guide for the Care and Use of Laboratory Animals, and experimental protocols were approved by the Research Ethics Committee of Shanghai Ocean University (No. SHOU‐DW‐2024‐023).

### Cell Culture and Treatment

MKC, MSpC, MBrC, and MLC cells were cultured in L‐15 medium (HyClone) supplemented with 15% fetal bovine serum (FBS; Gibco), 100 U mL^−1^ penicillin, and 100µg mL^−1^ streptomycin at 26 °C. BSR and HEK293 cells were cultured in DMEM medium (Gibco) supplemented with 10% FBS, 100 U mL^−1^ penicillin, and 100µg mL^−1^ streptomycin at 37 °C in 5% CO_2_.^[^ ^15^ ^]^

### Viral Infection

MKC or MSpC cells infected with VSV at a multiplicity of infection (MOI) of 0.1, 1, 10, or 100. MKC or MSpC cells were infected with SCRV at a multiplicity of infection (MOI) of 5. BSR cell was infected with rRABVs at the multiplicity of infection (MOI) of 0.1. After 1 h at 37 or 28 °C, the supernatant was discarded and cells were washed three times with PBS and then cultured in DMEM or medium 199 supplemented with 2% FBS (Gibco) at 34 or 28 °C.

### Virus Titration

To determine the viral titers of VSV, SCRV, and rRABV, MKC, MSpC, and BSR cells were infected with various dilutions of the viruses. Following a 1 h incubation at either 37 or 28 °C, the supernatant was decanted, and the cells were rinsed with PBS. The cells were then covered with DMEM supplemented with 2% Methylcellulose and incubated at 34 °C for 48 h or at 28 °C for 48 h. After incubation, the Methylcellulose was removed, and the cells were fixed and stained with a mixture of 0.1% crystal violet and 10% formalin in PBS under UV light. The staining process lasted for 4 h, after which the plates were rinsed with water to facilitate plaque counting.

### Plasmids Construction

To construct the Luc‐circNLRP12‐IRES‐WT, Luc‐circNLRP12‐IRES‐DEL1, Luc‐circNLRP12‐IRES‐DEL2 reporter vector, the IRES region of *M. miiuy* circNLRP12, as well as the base 1–87 in IRES region of circNLRP12 or the base 88–174 in IRES region of circNLRP12, were amplified using PCR and cloned into Luc2‐IRES‐report luciferase reporter vector (Geneseed Biotech). To construct circNLRP12‐P and circNLRP12‐P mutant plasmids, the full‐length circNLRP12 or mutant sequence was amplified by specific primers and cloned into pLC5‐circ vector (Geneseed Biotech), which contained a front and back circular frame to promote RNA circularization. The sequences of all primers are listed in Table S1 (Supporting Information).

### Prediction of the Secondary Structures of RNAs and Circularization in vitro

The secondary structures of circRnf103 were predicted at ^[NaCl]^ = 1.0 m and 37 °C by the UNAfold Web Server, and the optimal cyclization site of circNLRP12 was determined, and the linear‐circNLRP12 was synthesis from this cyclization site. For ligation experiments, a phosphate was in advance introduced to the 5′‐position of linear‐circNLRP12 by using T4 polynucleotide kinase (Beyotime) and T4 RNA Ligase 2 (Beyotime).^[^ ^16^ ^]^

### RNA Oligoribonucleotides

The sequences of RNA oligoribonucleotides are listed in Table S1 (Supporting Information).

### Cell Transfection

Transfection cells with siRNA (100nM) were performed in 24‐well plates using Lipofectamine RNAiMAX (Invitrogen), and cells were transfected with DNA plasmids (500 ng per well) or synthetic circRNA (500 ng per well) using Lipofectamine 3000 (Invitrogen) according to the manufacturer's instructions.

### Zebrafish Injection and Infection

Zebrafish, *Danio rerio* (4 months old) were maintained at 28 °C. The synthesized circRNA (10 µg) and Lipofectamine 3000 were mixed, then the mixture was left to stand at room temperature for 15 min, and the mixture was injected into the abdominal cavity of the zebrafish. After injecting circRNA into zebrafish for 24 h, VSV virus 10ul (MOI = 1) was injected intraperitoneally.

### RNA Extract and Quantitative Real‐Time PCR

Isolation of cytoplasmic and nuclear RNA from MKC cells, the Cytoplasmic & Nuclear RNA Purification Kit had been used (Norgen Biotek), and the content of circNLRP12 in the cytoplasm and nucleus was detected by PCR amplification. Total RNA was extracted using TRIzol Reagent from Invitrogen, and cDNA synthesis was carried out with the FastQuant RT Kit from Vazyme, which incorporates DNase treatment to remove genomic DNA contamination. Infect HEK293, EPC, or BSR cells with VSV, SCRV, and rRABV respectively, and then the corresponding circNLRP12 was transfected. Cells and culture medium were collected 48 h later, and after three freeze‐thaw cycles, PEG8000 was used to isolate and purify the virus from the sample. Finally, the virus RNA was extracted using TRIzol Reagent (Invitrogen). Gene expression analysis was conducted using SYBR Premix Ex TaqTM (Takara), and real‐time PCR was performed on a QuantStudio 3 system (Thermo Fisher Scientific). Primer sequences are displayed in Table S1 (Supporting Information).

### Dual‐Luciferase Report

To assess the activity of the circNLRP12‐IRES sequence, three plasmids encompassing distinct segments of the IRES element: full IRES sequence (IRES‐WT), the 1–87 nt of the IRES sequence (IRES‐DEL1), and the subsequent 88–174 nt (IRES‐DEL2) were constructed. The HEK293 cells were transfected with four constructs: Luc2‐IRES‐report, Luc2‐circNLRP12‐IRES‐WT, Luc2‐circNLRP12‐IRES‐DEL1, and Luc2‐circNLRP12‐IRES‐DEL2. Dual‐Luciferase Reporter was conducted at 24 and 48 h post‐transfection to evaluate the activity of each IRES variant.

### Antibody Generation and Western Blotting

Polyclonal antibodies against the VSV‐N, VSV‐P, VSV‐M, and NLRP12‐119aa polypeptides, which were produced by VSV and circNLRP12, were obtained by inoculating rabbits through (GenScript). Cellular and tissue lysates were generated by using 1 × SDS‐PAGE loading buffer. Collect natural protein samples using NP‐40 lysis buffer, and then add loading buffer without SDS. Proteins were extracted from cells and measured with the BCA Protein Assay kit (Vazyme), then subjected to SDS‐PAGE (8%) gel or native‐PAGE (8%) and transferred to PVDF (Millipore) membranes by semidry blotting (Bio‐Rad Trans Blot Turbo System). VSV‐N, VSV‐P, VSV‐M, and NLRP12‐119aa antibody were diluted at 1:200 (GenScript); anti‐Flag, anti‐HA, anti‐Myc, and anti‐Tubulin monoclonal antibody were diluted at 1:2000 (Sigma); and HRP‐conjugated anti‐rabbit IgG or anti‐mouse IgG (Beyotime) at 1:5000. The immunoreactive proteins were detected by using WesternBright^TM^ ECL (Beyotime). The digital imaging was performed with a cold CCD camera.

### Immunoprecipitation

For immunoprecipitation (IP) experiments, MSpC and HEK293 cells were seeded onto 10 cm^2^ plates and allowed to grow overnight. Subsequently, they were co‐transfected with 5 µg of the specified plasmids. After 48 h post‐transfection, the cells were washed three times with ice‐cold PBS. Following this, the cells were lysed with 500 µl of western and IP lysis buffer (Beyotime) supplemented with a protease inhibitor cocktail (Bitake) at 4 °C for 30 min on a rocking platform. The cellular debris was removed by centrifugation at 14 000 g for 15 min at 4 °C. The supernatant was then transferred to a new centrifuge tube and incubated with 50 µl of protein A + G beads (Sigma) and 1 µg of monoclonal anti‐Flag antibody (Sigma) or VSV antibody overnight at 4 °C with gentle rocking. On the subsequent day, the immunoprecipitated protein was pelleted by centrifugation at 2500 g for 5 min at 4 °C. The beads were washed five times with western and IP lysis buffer before being resuspended in 60 µl of 2 × SDS loading buffer. Both the immunoprecipitates and whole‐cell lysates (WCLs) were subjected to immunoblotting analysis.^[^ ^17^ ^]^

### RNA Immunoprecipitation

RIP experiments were conducted using the Magna RIP RNA‐Binding Protein Immunoprecipitation Kit from Millipore, adhering to the manufacturer's protocol. These assays were performed in MKC cells (≈2.0 × 10^7^) that had been transfected with circNLRP12. Post‐transfection at 24 h, VSV virus (MOI = 0.5) was added, and after an additional 24 h, the MKC cells were subjected to RIP assays using the Magna RIPTM RNA‐Binding Protein Immunoprecipitation Kit and an anti‐NLRP12‐119aa antibody, following the same protocol. RNA was extracted from the immunoprecipitated beads, and qPCR was employed to assess the expression levels of VSV‐leRNA.^[^ ^13b^ ^]^

### RNA Pulldown

The VSV‐leRNA with biotin labeling was first synthesized by using a Biotin RNA Labeling Kit (Beyotime). The RNA pulldown was conducted in MKC cells, and the specific steps were performed as described.^[^ ^18^ ^]^

### RNA Electrophoretic Mobility Shift

RNA‐EMSA was conducted according to the instructions of the EMSA/Gel Shift kit (Beyotime) to detect the interaction between leRNA of SCRV, VSV, RABV, and NLRP12‐119aa. The protein in *Escherichia coli* was expressed and purified.^[^ ^19^ ^]^ First, VSV‐N, VSV‐P, or NLRP12‐119aa proteins were mix purified with biotin‐labeled VSV‐leRNA and perform agarose gel electrophoresis. Then, gel electrophoresis on the following mixtures were conducted: 1) VSV‐N, VSV‐P, and biotin‐labeled VSV‐leRNA; 2) VSV‐N, VSV‐P, biotin‐labeled VSV‐leRNA, and NLRP12‐119aa; 3) VSV‐N, VSV‐P, biotin‐labeled VSV‐leRNA, and BSA. Finally, the electrophoresis results were documented using a gel imaging system. Similarly, the aforementioned procedures for SCRV and RABV were performed as well.

### LC‐MS Analysis

NLRP12‐119aa protein bands were manually excised from the gel and digested with sequencing‐grade trypsin (Promega). The digested proteins were analyzed using a QExactive mass spectrometer (Thermo). Fragment spectra were analyzed using the National Center for Biotechnology Information nonredundant protein database with Mascot (Matrix Science).^[^ ^20^ ^]^

### Statistical Analysis

Data are expressed as the mean ± SE from at least three independent triplicated experiments. Student's *t*‐test was used to evaluate the data. The relative gene expression data was acquired using the 2^‐∆∆CT^ method, and comparisons between groups were analyzed by one‐way analysis of variance (ANOVA) followed by Duncan's multiple comparison tests.^[^ ^21^ ^]^ A value of *p* < 0.05 was considered significant. All data, including immunoblots, are representative of three or more independent experiments.

## Conflict of Interest

The authors declare no conflict of interest.

## Author Contributions

W.Z. designed and performed experiments analyzed the data, performed bioinformatics analysis, and wrote the manuscript. W.Z. and X.Z. performed experiments and provided advice on experimental plans. W.Z., X.Z., T.Z., Q.L., and Y.Z. performed experiments. T.X. directed all the experiments and participated in experimental design, data analyses, interpretation, and manuscript writing.

## Supporting information



Supporting Information

## Data Availability

The data that support the findings of this study are available in the supplementary material of this article.
