# Supplementary material for: A Novel Protein NLRP12‐119aa that Prevents Rhabdovirus Replication by Disrupting the RNP Complex Formation
Source: Adv Sci (Weinh). 2025 Jan 4;12(12):2409953. doi: 10.1002/advs.202409953 (PMC11948066; doi:10.1002/advs.202409953)
Supplement: Supplementary file 1 — Supporting Information [file ADVS-12-2409953-s001.docx]

Supporting Information

A novel protein NLRP12-119aa that prevents rhabdovirus replication by disrupting the RNP complex formation

Weiwei Zheng^1^, Xiangxiang Zhu^1^, Tongtong Zhu^1^, Qiang Luo^1^, Yan Zhao^1^, Tianjun Xu^1,2,3,*^

1. *Laboratory of Fish Molecular Immunology, College of Fisheries and Life Science, Shanghai Ocean University, Shanghai, China*
2. *Laboratory for Marine Biology and Biotechnology, Qingdao Marine Science and Technology Center, Qingdao, China*
3. *Marine Biomedical Science and Technology Innovation Platform of Lin-gang Special Area, Shanghai, China*

*Corresponding author: Dr. Tianjun Xu

E-mail: tianjunxu@163.com

# 1. Supplementary Figure


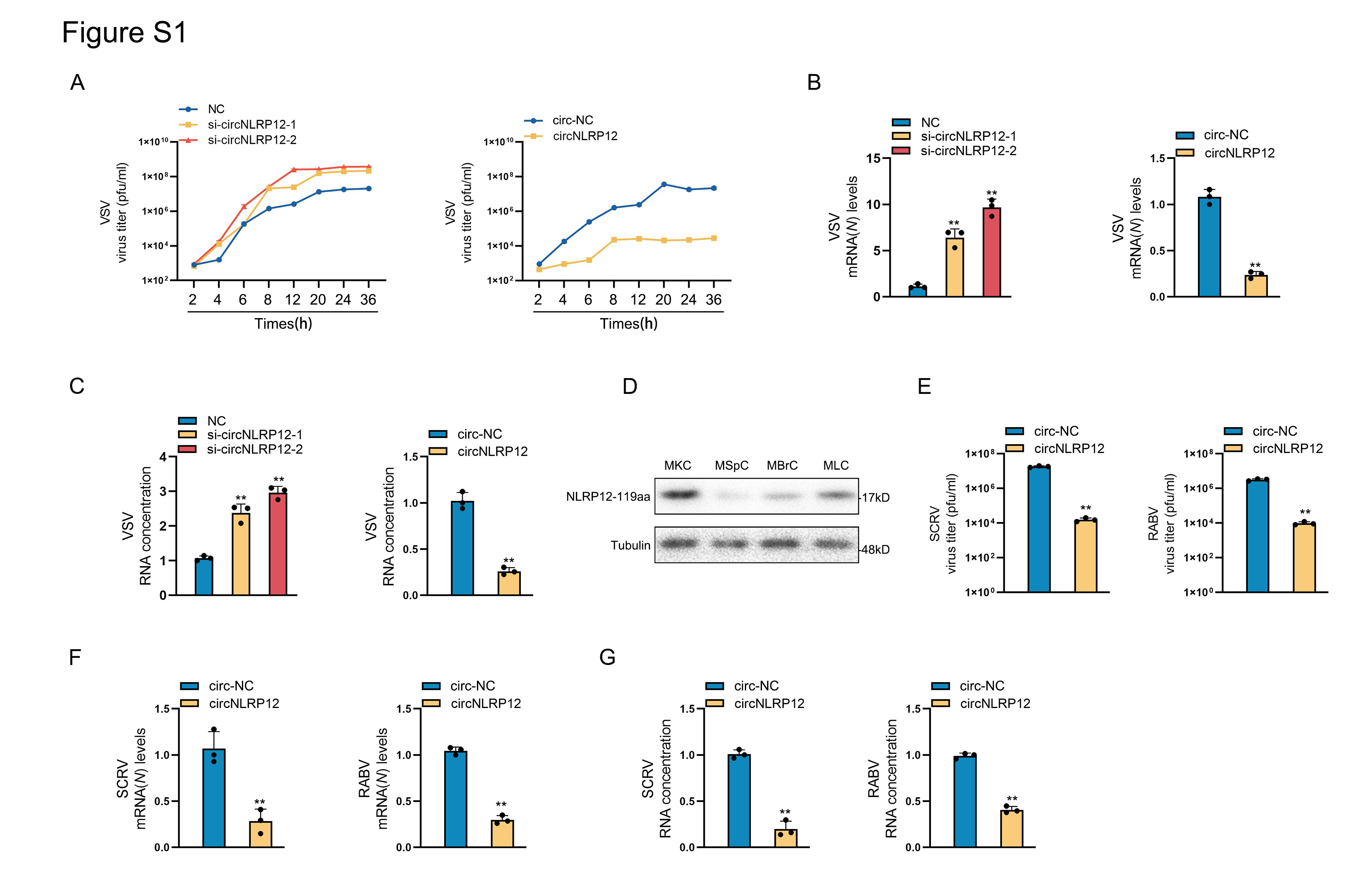


**Figure S1. Anti-rhabdoviral activity of NLRP12-119aa.** (A) Transfect si-circNLRP12 or circNLRP12 into MKC cells infected with VSV, and then measure the VSV virus titers at various time points and plot the one-step growth curve of the virus (n=3). (B and C) Level of VSV mRNA (B) and virus total RNA (C) were detected after transfected with si-circNLRP12 in MKC cells with VSV infection or transfected with circNLRP12 in MSpC cells with VSV infection (n=3). (D) Expression of NLRP12-119aa protein in MKC, MSpC, MBrC, and MLC cells. (E) Levels of SCRV and RABV viral titer were detected after being transfected with circNLRP12 in MSpC or BSR cells with SCRV or RABV infection, respectively. (E and F) Levels of SCRV and RABV mRNA (F) and virus total RNA (G) were detected after transfected with circNLRP12 in MSpC or BSR cells with SCRV or RABV infection, respectively. All data represent the means ± SE from three independent triplicate experiments. *, *p*<0.05; **, *p*<0.01.

# 2. Supplementary Table

**Table S1.** RNA oligoribonucleotides and PCR primer information in this study.

| **RNA oligoribonucleotides** | **Sequences (5’-3’)** |
| --- | --- |
| NC | CUGGUGAUACUAAGCGAAGTT |
| si-circNLRP12-1 | CUGGUGAUACUAAGGCUUUTT |
| si-circNLRP12-2 | GGUGAUACUAAGGCUUUUUTT |
| VSV-leRNA(1-47) | TGCTTCTGTTTGTTTGGTAATAATAGTAATTTTCCGAGTCCTCTTTGAAATTGTCATTAGTTT |
| VSV-leRNA(1-18) | TGCTTCTGTTTGTTTGGT |
| VSV-leRNA(19-47) | AATAATAGTAATTTTCCGAGTCCTCTTTGAAATTGTCATTAGTTT |
| VSV-leRNA(1-23) | TGCTTCTGTTTGTTTGGTAATAA |
| VSV-leRNA(24-47) | TAGTAATTTTCCGAGTCCTCTTTGAAATTGTCATTAGTTT |
| SCRV-leRNA(1-70) | TGCTCTTTTTTCTTTGGTTATATGTCTAATAGTTCTAATAACCTAAAGAAAGAGAGCTGTAAGTTTTACA |
| SCRV-leRNA(1-18) | TGCTCTTTTTTCTTTGGT |
| SCRV-leRNA(19-70) | TATATGTCTAATAGTTCTAATAACCTAAAGAAAGAGAGCTGTAAGTTTTACA |
| SCRV-leRNA(1-35) | TGCTCTTTTTTCTTTGGTTATATGTCTAATAGTTC |
| SCRV-leRNA(36-70) | TAATAACCTAAAGAAAGAGAGCTGTAAGTTTTACA |
| RABV-leRNA(1-58) | TGCGAATTGTTGTTTTGGTTTCTTCTTCGTCTGTCGCAGTCAACGTTTCGTTTTTACA |
| RABV-leRNA(1-19) | TGCGAATTGTTGTTTTGGT |
| RABV-leRNA(20-58) | TTCTTCTTCGTCTGTCGCAGTCAACGTTTCGTTTTTACA |
| RABV-leRNA(1-29) | TGCGAATTGTTGTTTTGGTTTCTTCTTCG |
| **Primer** | **Sequences (5’-3’)** |
| circNLRP12-P-1F | AATATTTCTTCTTTCGAATTCTAATACTTTCAGGCTTTTTAAGACAAACCTACAAAAGG |
| circNLRP12-P-1R | TGGAGTTGTTAGCTAGGATCCAGTTGTTCTTACCTTAGTATCACCAGTTTACACTGAGGTTT |
| Flag-circNLRP12-P-1F | TACAAAGACGATGACGACAAGTGAGACAGTCCTCATGCTTAAAGTATAG |
| Flag-circNLRP12-P-1R | GTCGTCATCGTCTTTGTAGTCCCTCAGTGTGTCCACTTTGCAG |
| Flag-circNLRP12-ATG-mut-P-1F | AAAATCTCCTTCCAGCCTGAAATGCTGCTCAT |
| Flag-circNLRP12-ATG-mut-P-1R | GGCTGGAAGGAGATTTTTAAACAGTTCTCACAGGTTGTGTTG |
| Flag-circNLRP12-IRES-mut-P-1F | GCGTTAGTCAGGTATGTGTCCTTTGAAGATGTGAAGACT |
| Flag-circNLRP12-IRES-mut-P-1R | CACATACCTGACTAACGCACTTTACCCAGGACC |
| Flag-circNLRP12-1F | TAATACGACTCACTATAGGGGCTTTTTAAGACAAACCTACAAAAGG |
| Flag-circNLRP12-1R | CTTAGTATCACCAGTTTACACTGAGGTTT |
| IRES-WT-1F | GCGTGCTAGCCCGGGCTCGAGACCATTTAGATAAGTTTGTCTGACTCCT |
| IRES-WT-1R | CAGTACCGGAATGCCAAGCTTCACTCGATAGTCACTGAAATCTCATTT |
| IRES-DEL-1-1F | TCAGCCAAGCTTGGCATTCCGGTACTGTTGGTA |
| IRES- DEL-1-1R | AATGCCAAGCTTGGCTGAGCAGTGTGTTATCCCA |
| IRES- DEL-2-1F | GGCTCGAGACTTGGAAAAGAATGGGCCAAAGTG |
| IRES- DEL-2-1R | TTTTCCAAGTCTCGAGCCCGGGCTAGCACGCGT |
| SCRV-N-1F | GACGATGACGACAAGAAGCTTATGGAACACCAAATCATCAGGAG |
| SCRV-N-1R | TGATGGATATCTGCAGAATTCTCACAAAGCTTGGTGCTTCAGC |
| SCRV-P-1F | CCCAAGCTTATGGCAAAACCAACTTTCCAGA |
| SCRV-P-1R | CCGGAATTCTTACCGAATCACCTCGAGAGGG |
| VSV-N-1F | GACGATGACGACAAGAAGCTTATGGCTCCTACAGTTAAGAGAATCATT |
| VSV-N-1R | TGATGGATATCTGCAGAATTCTCACTTGTCAAACTCTGCCTTGG |
| VSV-P-1F | GACGATGACGACAAGAAGCTTATGGACAGTATTGATCGGCTCAA |
| VSV-P-1R | TGATGGATATCTGCAGAATTCTTAGAGACGATACTTCATTCTTGCTTG |
| RABV-N-1F | GACGATGACGACAAGAAGCTTAAATGTAACACCCCTACAATGGATG |
| RABV-N-1R | TGATGGATATCTGCAGAATTCTTTTTTTCATGATGGATATACACAATCT |
| RABV-P-1F | GACGATGACGACAAGAAGCTTACCATCCCAAGTATGAGCAAGATC |
| RABV-P-1R | TGATGGATATCTGCAGAATTCTTTTTTTCATGTCGACTCCATGA |
| VSV-N-RT-1F | ACGACTCCATTATTCAGC |
| VSV-N-RT-1R | CTCCTCTCTCTTTCCGAT |
